# Ire1 supports normal ER differentiation in developing *Drosophila* photoreceptors

**DOI:** 10.1242/jcs.180406

**Published:** 2016-03-01

**Authors:** Zuyuan Xu, Madhusudana Rao Chikka, Hongai Xia, Donald F. Ready

**Affiliations:** Department of Biological Sciences, Purdue University, West Lafayette, IN 47907, USA

**Keywords:** *Drosophila*, ER, Photoreceptor, Ire1

## Abstract

The endoplasmic reticulum (ER) serves virtually all aspects of cell physiology and, by pathways that are incompletely understood, is dynamically remodeled to meet changing cell needs. Inositol-requiring enzyme 1 (Ire1), a conserved core protein of the unfolded protein response (UPR), participates in ER remodeling and is particularly required during the differentiation of cells devoted to intense secretory activity, so-called ‘professional’ secretory cells. Here, we characterize the role of Ire1 in ER differentiation in the developing *Drosophila* compound eye photoreceptors (R cells). As part of normal development, R cells take a turn as professional secretory cells with a massive secretory effort that builds the photosensitive membrane organelle, the rhabdomere. We find rough ER sheets proliferate as rhabdomere biogenesis culminates, and Ire1 is required for normal ER differentiation. Ire1 is active early in R cell development and is required in anticipation of peak biosynthesis. Without Ire1, the amount of rough ER sheets is strongly reduced and the extensive cortical ER network at the rhabdomere base, the subrhabdomere cisterna (SRC), fails. Instead, ER proliferates in persistent and ribosome-poor tubular tangles. A phase of Ire1 activity early in R cell development thus shapes dynamic ER.

## Abstract (precis)

**Highlighted Article:** Developing *Drosophila* photoreceptors dynamically remodel ER to meet changing cell needs; success requires the conserved multifunctional ER regulator Ire1.

## INTRODUCTION

Cells dynamically remodel endoplasmic reticulum (ER) to meet changing physiological demand (Borgese et al., 2006; Federovitch et al., 2005). For example, and typical of so-called ‘professional’ secretory cells generally, rough ER (rER) is amplified over threefold as B cells differentiate into antibody-secreting plasma cells (Wiest et al., 1990); conversely, when secretory demand is diminished, as when estrogen is withdrawn from *Rana* (brown frog) hepatocytes previously stimulated to amplify ER for vitellogenin secretion, rER returns to basal levels (Herbener et al., 1983). The pathways that mediate ER expansion and regression are incompletely understood, but include inositol-requiring enzyme1, Ire1, a conserved core protein of the unfolded protein response (UPR), a network that promotes ER homeostasis (Ron and Walter, 2007). Ire1 transduces ER stress through endonuclease and kinase activity: the Ire1 endonuclease activity excises a stress-sensitive intron from mRNA encoding X-box-binding protein 1, Xbp1, switching the open reading frame to encode a potent transcriptional activator of hundreds of genes that support proteostasis (Acosta-Alvear et al., 2007); independently of Xbp1, Ire1 endonuclease further degrades multiple mRNAs and pre-miRNAs through the regulated Ire1-dependent decay of mRNA (RIDD) pathway, regulating numerous targets controlling cell physiology and fate (Coelho and Domingos, 2014; Hollien and Weissman, 2006; Maurel et al., 2014). Notably, Ire1 activity is required early in B cell differentiation to build secretory capacity in preparation for the high-level antibody secretion of mature plasma cells (Zhang et al., 2005) and thyrocytes exposed to thyrocyte stimulating hormone increase the amount of ER in anticipation of thyroglobulin secretion (Christis et al., 2010). Conversely, Ire1 inhibition compromises dexamethasone-induced ER expansion in a cell culture model of pancreatic development (Cross et al., 2012), and hepatocytes lacking Ire1 show reduced rER (Zhang et al., 2011). Ire1 thus acts beyond coping with ER stress and includes normal developmental ER programming (Wu and Kaufman, 2006).

ER is organized into distinct structural and functional domains (Baumann and Walz, 2001) by multiple proteins and forces (Westrate et al., 2015). Notably among ER-shaping proteins are reticulons, proteins that insert hairpin-like transmembrane domains into the cytosolic ER membrane and impose strong curvature at edges of flat cisternae and along tubules (Voeltz et al., 2006). How forces that shape the ER are balanced to achieve required form is under active investigation, but Ire1 is again implicated by observations in yeast that show although Ire1 mutants have normal ER morphology in the absence of ER stress, when stressed they produce tangled knots of irregular, reticulon-rich ER tubules (Schuck et al., 2009).

Developing *Drosophila* photoreceptors (R cells) are a favorable venue for studies of ER dynamics. Late in R cell differentiation, as professional secretory cells, they build and support an enormous, rhodopsin-rich photosensory plasma membrane organelle, the rhabdomere, which signals through Ca^2+^ influx, thus drawing heavily on core ER functions: phospholipid and membrane protein biosynthesis and Ca^2+^ homeostasis. Like the rhabdomere it supports, R cell ER is greatly amplified and anatomically stereotyped. Adult R cell ER shows canonical nuclear envelope and peripheral domains, the later including sparse rER cisternae connected by tubules to the elaborate network of cortical ER tubules apposed to the rhabdomere base, the subrhabdomeric cisternae (SRC) (Baumann and Walz, 2001; Matsumoto-Suzuki et al., 1989). The SRC regulates cytosolic Ca^2+^ (Walz and Baumann, 1995) and participates in phospholipid and membrane protein transport to the rhabdomere (Masai et al., 1997; Suzuki and Hirosawa, 1991; Vihtelic et al., 1993). Dynamic R cell ER reorganization is evident in the response to stress, for example, in the expansion of rER cisternae in NinaA mutants, which are deficient in an ER chaperone required for proper rhodopsin folding (Colley et al., 1991; Kurada and O'Tousa, 1995), as well as in SNARE-encoding Sec22 mutants, which compromise ER–Golgi trafficking (Zhao et al., 2015), in the proliferation of abnormal ER in dPOB (also known as EMC3) mutants, which misfold multipass membrane proteins (Satoh et al., 2015), and in the response to light stress (Weiss and Minke, 2015). Recently, Ire1 loss has been shown to reduce expression of the R cell secretory proteins Eyes shut (also known as Spacemaker) and rhodopsin-1 (Rh1, also known as NinaE), and RIDD downregulation of fatty acid transporter, Fatp, mRNA has been shown to facilitate Rh1 delivery to developing rhabdomeres (Coelho et al., 2013). Here, we examine the role of Ire1 in R cell ER differentiation.

## RESULTS

### R cell rough ER proliferates during peak biosynthetic load

**Fig. 1.**
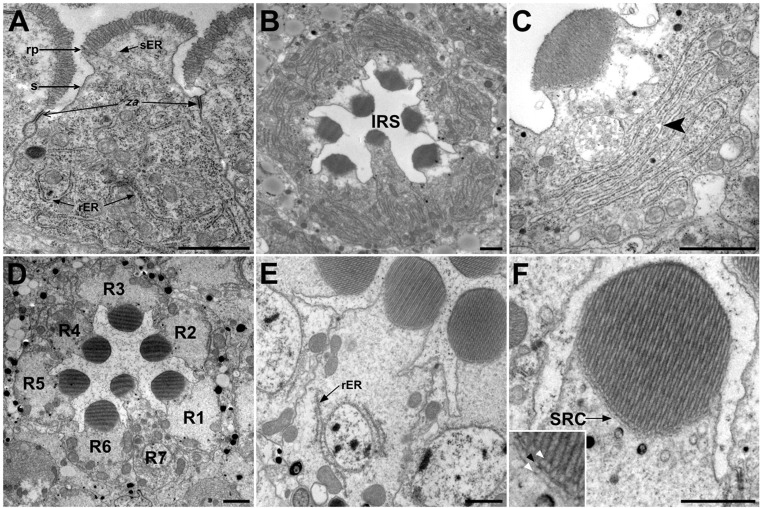
**R cell rER proliferates at the peak biosynthetic demand.** (A) Rhabdomere development begins in mid-pupal life with the differentiation of the apical plasma membrane into two distinct domains, the rhabdomere primordium (rp), a fringe of stubby microvilli and the stalk (s), a collar of unamplified membrane between zonula adherens (za) junctions and the rhabdomere. Mid-pupal R cell cytoplasm shows occasional rER sheets throughout the cytoplasm and disordered tubules of smooth ER approaching the developing rhabdomere. sER, smooth ER. (B,C) At 85% pupal development, stacked rER sheets dominate R cell cytoplasm. Sheets merge to share a common lumen (arrowhead). (D,E) In adult R cells rER is sparse and often located near the nucleus and towards the basal pole of the cell. (F) In adult R cells, an extensive tubular network of cortical ER, the SRC, is applied to the base of the rhabdomere. In the inset, white arrowheads mark the ∼130-nm span where dark loops that mark the cytoplasmic termini of plasma membrane microvilli meet the SRC. Enhanced density at SRC–plasma-membrane contacts (black arrowhead) suggests a specialized nexus. Scale bars: 1 μm.

Rhabdomere development begins in mid-pupal life with the differentiation of the apical plasma membrane into two distinct domains, the rhabdomere primordium, a fringe of stubby microvilli, and the stalk, a collar of unamplified supporting membrane between the zonula adherens junctions and the rhabdomere. Although mid-pupal R cells are actively growing, they have yet to begin the massive secretory endeavor that builds rhabdomeres. At this stage, R cell ER is ‘generic’, presenting rER cisternae snaking throughout the cytoplasm without apparent order; tubules of smooth ER commonly approach the developing rhabdomere, but lack the organization seen later in the SRC (Fig. 1A). Rhodopsin synthesis begins in the last quarter of pupal development, and by 85% pupal development copious Rh1 is being delivered to rapidly elongating rhabdomere microvilli (Kumar and Ready, 1995). ER proliferation accompanies this massive secretory push; rER is prominent (Fig. 1B,C). In adult R cells, no longer dominated by rhabdomere differentiation, rough ER cisternae are seen in thin sections as sparse rER cisternae, often near the nucleus and the basal pole of the cell (Fig. 1D,E); cortical ER of the SRC is extensive across the base of the rhabdomere (Fig. 1F).

### Ire1 is active during early R cell differentiation

In view of the role of Ire1 in secretory cell differentiation, we asked whether it was likewise active in highly secretory developing R cells. Following the strategy of Iwawaki et al. (2004), we designed a Myc–Xbp1–GFP (MXG) reporter, in which Ire1-mediated excision of the Xbp1 stress-sensitive intron brings a C-terminal GFP into the open reading frame of spliced Xbp1 (Xbp1s); an N-terminal Myc tag signals reporter expression. Similar *Drosophila* Ire1 reporters have been described, using full-length Xbp1 (Sone et al., 2013; Souid et al., 2007) and using Xbp1 mRNA truncated close to the excision site (Ryoo et al., 2007); a full-length reporter has been shown to be more sensitive than the truncated form (Sone et al., 2013). To validate MXG, we expressed it in third-instar eye discs and treated them with dithiothreitol (DTT), which blocks disulfide bond formation and thus protein folding, a standard protocol to induce UPR (Ryoo et al., 2007; Souid et al., 2007). Anti-Myc antibody staining confirmed that MXG expression begun about 12 rows behind the morphogenetic furrow, and GFP was strongly expressed in DTT-treated discs (Fig. S1).

**Fig. 2.**
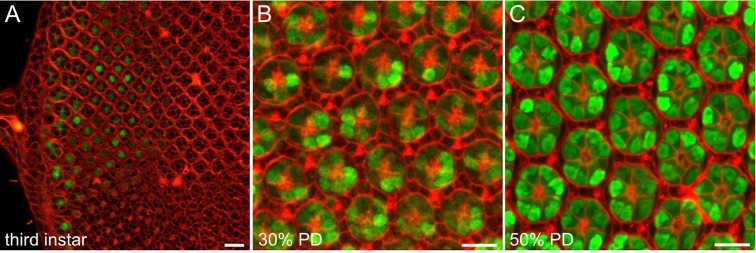
***In vivo* imaging shows Ire1 activity early in R cell differentiation.** (A) In a live third-instar eye disc, FM4-64 (red) marks cell outlines, whereas MXG reports Ire1 endonuclease through GFP (green) expression in R8 cells at the posterior of the disc. R cell nuclei occupy stereotyped planes in the developing third-instar eye (Tomlinson, 1985) and the R8 nuclear plane at the posterior of the disc is highlighted by GFP. Anterior is to the right and the morphogenetic furrow is close outside the frame. (B) In a confocal *z*-stack composite of a live 30% pupal development eye, FM4-64 (red) shows the immature, unresolved apical tiling of interommatidial cells in a single optical section overlaid on a maximum intensity projection of optical sections of underlying R cell nuclei; GFP (green) shows Ire1 activity in many R cells. (C) A composite confocal *z*-stack image of a live 50% pupal development eye. A single optical plane FM4-64 (red) shows the resolved interommatidial cell lattice image and ‘wagon wheel’ R cell organization overlaid on maximum projection through underlying R cells. All R cells express GFP (green). Scale bars: 10 μm.

*In vivo* imaging using MXG detects Ire1 endonuclease early in R cell differentiation, approximately 1 day after R cell fates are specified along the morphogenetic furrow that marks the leading edge of retinal differentiation. R8, the first cell specified and founder for the recruitment of subsequent cells into a stereotyped ommatidial cluster, occupies a central position in the cluster and began to express GFP beginning approximately 12 rows behind the furrow (Fig. 2A). Notably, the complex cell–cell signaling and choreography that builds the mature eight-cell ommatidial cluster does so without evident Ire1 activity. As retinal development continues in early pupae, additional R cells expressed GFP (Fig. 2B); apart from R8, the first specified R cell, expression of GFP in other R cells was without apparent order, suggesting that Ire1 activity is not closely coupled to the order in which R cells are recruited. By mid-pupal life, all R cells expressed GFP, and varied levels of both nuclear and cytoplasmic MXG were seen (Fig. 2C). Ire1 activity has previously been reported in developing R cells, beginning later, in mid-pupal development (Coelho et al., 2013), likely reflecting use of the truncated Xbp1 reporter. These results show Ire1 endonuclease is active early in R cell differentiation, well before ER proliferation and the massive secretion that builds the rhabdomere.

### Ire1 loss disrupts ER anatomy

**Fig. 3.**
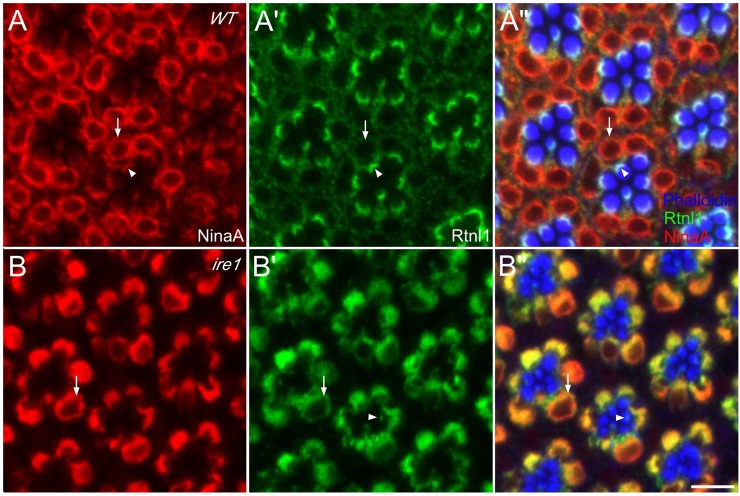
**Ire1 loss disrupts R cell ER.** (A) In wild-type adult R cells, NinaA (red) highlights the nuclear envelope (arrow) and the SRC (arrowhead). NinaA in the SRC marks a crescent at the rhabdomere base. NinaA-stained tubules connecting the SRC and nuclear envelope are captured in part traversing the optical plane and can appear dim. (A′) Rtnl–GFP (green) highlights the SRC. ER marked by Rtnl1–GFP extends throughout cytoplasm but is notably absent from the nuclear envelope. (A″) Phalloidin (blue) binds the central F-actin filament of each microvillus, staining rhabdomeres. F-actin in the terminal web at the rhabdomere base overlaps with the SRC, seen as a white crescent. (B–B″) R cell growth compresses interommatidial cells to a thin hexagonal grid. In Ire1^−/−^ R cells, NinaA (red), Rtnl1–GFP (green) and phalloidin (blue), show abnormal ER and rhabdomeres. NinaA and Rtnl1–GFP colocalize in dense cytoplasmic accumulations. The nuclear envelope outline (arrow) is lost against the virtually solid cytoplasmic NinaA stain; unlike wild-type, mutant nuclei do not lie on a well-defined plane. The SRC (arrowhead) is reduced to a small remnant at the base of reduced and dysmorphic rhabdomeres. Scale bars: 5 μm.

As Ire1 is active in developing R cells, we asked whether it was also required for normal ER differentiation. An insertion mutation, *Ire1^f02170^*, is larval lethal (Ryoo et al., 2007). To circumvent lethality, we made eye mosaics containing mutant R cells in viable, heterozygous animals and examined ER organization using confocal immunofluorescence for the ER chaperone NinaA and GFP-tagged Rtnl1, the single ER-tubule-associated *Drosophila* reticulon (Wakefield and Tear, 2006). In wild-type adult R cells, NinaA localized throughout the ER and was prominent in the nuclear envelope and SRC (Fig. 3A). Rtnl1–GFP localized throughout peripheral ER generally and highlighted the SRC, where cortical ER tubules applied to the base of the rhabdomere cylinder stack signal in the *z*-axis; Rtnl1–GFP was notably absent from low curvature nuclear envelope (Fig. 3A′). Staining the central F-actin filament threading each rhabdomere microvillus with phalloidin highlighted the rhabdomere, encompassing over 90% of the plasma membrane and the product of intense secretory activity. F-actin at the rhabdomere base, the rhabdomere terminal web, overlapped the SRC (Fig. 3A″).

The ER was profoundly disrupted in developing Ire1 mutant R cells. Optical sections showed that NinaA and Rtnl1 colocalized in dense cytoplasmic aggregates and only small remnants of SRC remained (Fig. 3B,B′). Phalloidin staining showed that rhabdomeres and their underlying terminal web were severely reduced (Fig. 3B″). Ire1 mutant R cells were strongly growth deficient; reduced cross-sections of mutant ommatidia were plain in optical sections as broad, dark separations between ommatidia (Fig. 3B) and in electron microscopy thin sections there were seen as expanded profiles of interommatidial cells (Fig. S2). Measured using confocal and electron microscopy, mutant ommatidial cross-sections ranged from 52–74% of wild type (Table S1). Ommatidial length, measured using live ommatida dissociated from wild-type and whole eye mosaics, was reduced by ∼40% (Table S2). As reported previously (Coelho et al., 2013), the inter-rhabdomeral space (IRS), which is normally opened by copious R cell apical secretion of the extracellular matrix proteoglycan Eyes shut (Husain et al., 2006; Zelhof et al., 2006), is strongly reduced in Ire1 mutant ommatidia (Fig. S2). Thus, in addition to disrupting ER organization and rhabdomere morphogenesis, Ire1 loss compromises overall R cell growth.

### Ire1 loss causes progressive failure of normal ER organization and extreme tubulation

**Fig. 4.**
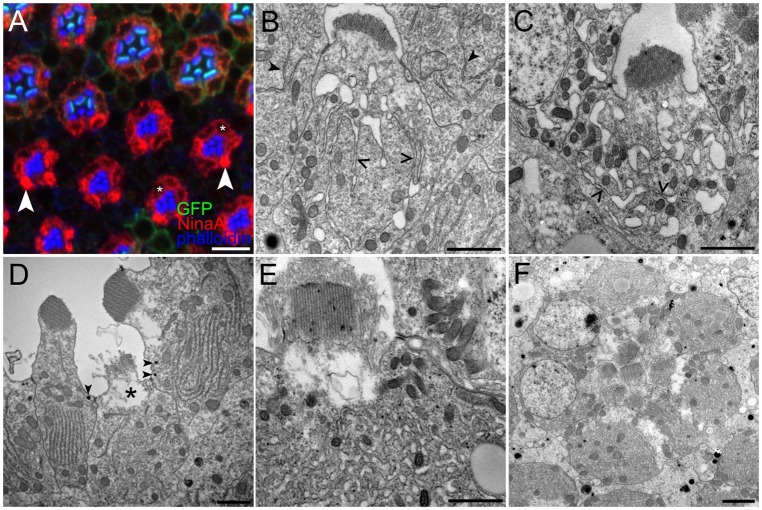
**Ire1 loss degrades ER structure.** (A) In a mosaic eye at ∼70% pupal development, Ire1 mutant cells, lacking myristoylated GFP (myr.GFP; green), show abnormal NinaA (red) cytoplasmic accumulation. In wild-type cells, membrane-localized myr.GFP highlights stubby microvilli sitting atop phalloidin-stained (blue), actin-rich apical cytoplasm. Some mutant cells show abnormal, dense NinaA accumulations (arrowheads), whereas others appear normal (asterisks). Phalloidin shows all mutant rhabdomeres are stunted relative to wild-type cells. (B) At ∼65% pupal development, R cell dysmorphic ER becomes evident as dilation of normally small loops at cisternae edges. Apparently normal, ribosome-studded rER cisternae are continuous with dilated loops (open arrowheads). Note normal rER in neighboring mutant R cells (solid arrowheads). Rhabdomere structure appears normal for this stage. (C) At 85% pupal development, ER is dilated and short runs of rER are rare (open arrowheads). Mutant rhabdomeres are reduced and irregular relative to wild type. (D) In an mosaic ommatidium at 85% pupal development, a mutant R1 (asterisk), marked by the loss of small ommochrome pigment granules (arrowheads) present in wild-type R cells, fails to form the stacked rER seen in flanking wild-type R7 and R2 cells (as judged by the presence of small pigment granules; arrowheads). The mutant rhabdomere is strongly degenerated. (E) At 90% pupal development, tubular, dilated ER fills R cell cytoplasm. Rhabdomeres are highly abnormal, here showing a core of closely stacked microvilli surrounded by a halo of irregular, loosely packed microvilli. (F) A cross-section through a fully mutant adult ommatidium shows abnormal ER virtually filling R cell cytoplasm. Mutant R cells are smaller than wild type, presenting reduced cross-sectional profiles. R cell nuclear envelope appears normal. Rhabdomeres are highly degenerate. Scale bars: 10 μm (A), 1 μm (B–F).

ER organization in Ire1 mutant R cells diverges from wild type at ∼70% pupal development as dense cytoplasmic NinaA aggregates appeared in some cells; at this stage wild-type rhabdomeres took on an elongated, flattened appearance, whereas mutant rhabdomeres retained a smaller, rounded appearance (Fig. 4A). ER distortion was plain in the electron microscope as irregular, ribosome-poor dilations communicating with normal-appearing rER cisternae (Fig. 4B). By 80% pupal development, mutant R cell cytoplasm was dominated by abnormal dilated ER; small patches of ribosome-studded rER were continuous with irregular dilations (Fig. 4C). In mosaic eyes, mutant R cells did not assemble the rER stacks typical of wild-type neighbors (Fig. 4D). By 95% pupal development, tubular ER filled mutant R cell cytoplasm and rhabdomeres were strongly degenerate (Fig. 4E). Contrary to the reduction of rER seen in wild-type R cells as they shift from a developmental secretory mode to a mature photosensory role, abnormal tubulated ER persisted in adult mutant R cells (Fig. 4F).

**Fig. 5.**
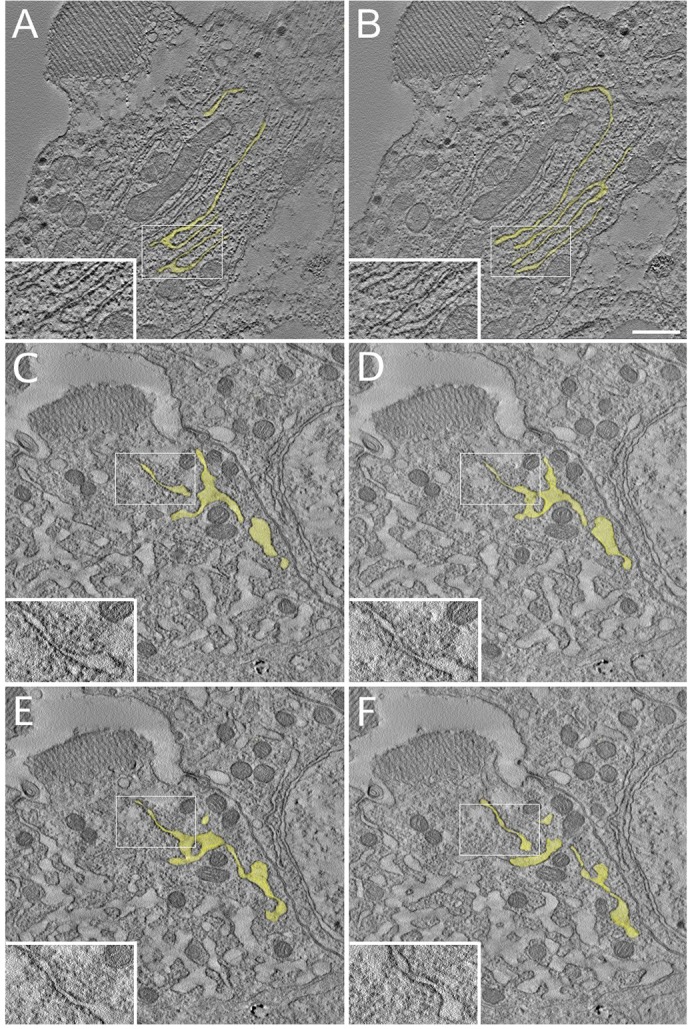
**R cell EM tomography at peak biosynthesis.** In a wild-type cell at 85% pupal development, tomographic 2.1 nm slices at (A) 0 nm and (B) 54.6 nm show that rER sheets extend on the long axis of the R cell and share a common lumen (yellow) as parallel sheets merge. Insets (magnified areas of the boxed regions) show four parallel sheets that merge along their lateral edges. In an 85% pupal development Ire1 mutant R cell, tomographic slices at (C) 0 nm, (D) 27.4 nm, (E) 75.5, (F) 121.8 nm show isolated profiles demonstrating that they share a common lumen (yellow). Small patches of sheet-like ribosome-studded ER communicate with irregular, ribosome-poor ER dilations (insets). Scale bar: 500 nm.

A hallmark of ER topology, the stacked, parallel rER sheets of R cells at peak biosynthesis were seen in electron tomograms to share a common lumen, joining adjacent sheets at their lateral edges (Fig. 5A,B). Although mutant ER is highly dysmorphic at this stage, lumen continuity was preserved; tomography showed small rER sheets communicating with irregular, bulgy tubular ER and, hence, Ire1 loss does not fragment ER (Fig. 5C–F). So expansive was mutant ER, that electron micrographs showed a figure-ground reversal, that is, islands of darker, granular cytoplasm in a lake of pale ER lumen. The near total occupation of mutant cytoplasm by tubular ER in electron micrographs is reasonably the ultrastructural correlate of the abnormal, dense NinaA and Rtnl1–GFP aggregates seen in confocal immunofluorescence. R cells thus require Ire1 to proliferate normally organized rER and amplify secretory capacity.

### Ire1 loss severely attenuates Rh1 accumulation and rhabdomere morphogenesis

**Fig. 6.**
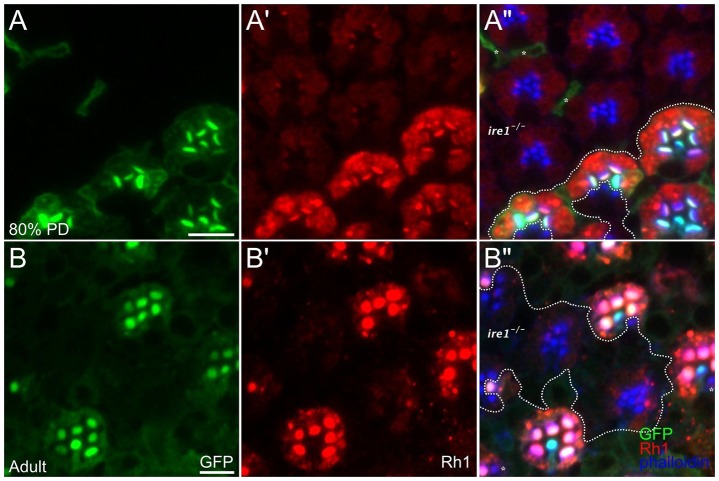
**Ire1 loss compromises Rh1 levels and rhabdomere differentiation.** (A–A″) Along the clonal boundary (dashed line) between *ire1*^−/−^ and *ire1*^+/−^ cells in an 80% pupal development mosaic eye, wild-type R1–R6 (myr.GFP-positive) cells show strong cyotplasmic Rh1 (red) and early Rh1 delivery to the rhabdomere (blue). Mutant rhabdomeres are stunted relative to the broad and flattened, stage-specific rhabdomeres of wild-type R cells. Asterisks in A″ mark three wild-type ‘outlier’ pigment cells isolated from the main body of the wild-type clone. (B) In a mosaic adult eye, wild-type rhabdomeres present mature, round profiles, fully-loaded with Rh1. In mutant R cells, Rh1 is barely detectable and rhabdomeres are severely stunted. Scale bar: 5 μm.

Wild-type R1–R6 cells begin Rh1 production at ∼70% pupal development, which is first evident as light, diffuse cytoplasmic immunofluorescence (Kumar and Ready, 1995; Satoh et al., 2005). By 80% pupal development, R cells are fully engaged with Rh1 synthesis, showing strong cytoplasmic staining and early delivery to the developing rhabdomere. Side-by-side comparison of Rh1 levels in mutant and wild-type R cells along clonal borders showed that Ire1 mutant R cells had low levels of cytoplasmic Rh1, weak Rh1 accumulation at the rhabdomere base and no evident transfer to the rhabdomere (Fig. 6A–A″). Unlike the abundant accumulation of Rh1 in Rab11 mutant R cells, where trans-Golgi network (TGN)–rhabdomere delivery fails, or the ample Rh1 accumulated in the R cell ER when ER-Golgi transfer is prevented by vitamin A deprivation (Ozaki et al., 1993; Satoh et al., 2005), Rh1 levels in Ire1 mutant R cells were strongly reduced from the outset, suggesting that reduced Rh1 synthesis or rapid degradation of newly-synthesized Rh1 by ER-associated degradation (ERAD) (Kang and Ryoo, 2009), rather than blockage of Rh1 transport was the root cause of the phenotype. In contrast to the broad, flattened rhabdomeres that characterized wild-type R cells, mutant rhabdomeres were stunted (Fig. 6A″). Given that rhabdomeres completely lacking Rh1 are approximately normal in size at this time (Kumar and Ready, 1995), reduced rhabdomeres of Ire1 mutant R cells reasonably reflect broad attenuation of rhabdomere-building secretion. Rhabdomeres of wild-type adult R cells were fully Rh1-loaded, with a mature, round profile; in adult Ire1 mutants, Rh1 was barely detectable in severely reduced rhabdomeres (Fig. 6B–B″). As Ire1 has been shown to compromise Rh1 delivery and rhabdomere morphogenesis independently of Xbp1 (Coelho et al., 2013), we used whole eye mosaics to ask whether the ER shaping activity of Ire1 is likewise Xbp1-independent and found that it was (Fig. S3).

### ER collapse upon Ire1 loss is temperature dependent with a sensitive period anticipating peak biosynthesis

**Fig. 7.**
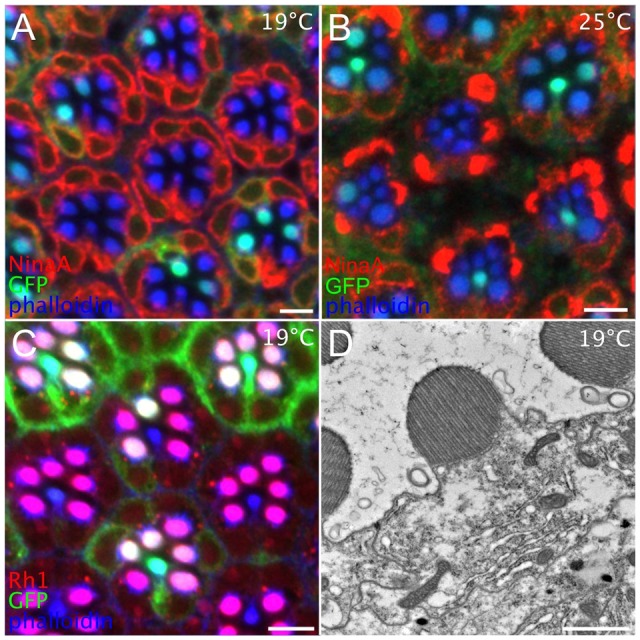
**Temperature shifts define a crucial period for Ire1 activity.** (A) In a mosaic eye of an animal raised at 19°C, *ire1*^−/−^ cells, marked by GFP (green) loss, show normal ER and rhabdomeres. Red, NinaA; blue, phalloidin. (B) Mosaics raised at 25°C, show dense cytoplasmic NinaA accumulation and reduced rhabdomeres. (C) In a mosaic raised at 19°C, *ire1*^−/−^ cells show normal Rh1 (red) delivery to ‘purple’ (red+blue) R1–R6 rhabdomeres, comparable to ‘white’ tinged (green+red+blue) *ire1*^+/−^ rhabdomeres. Lacking Rh1, central R7 rhabdomeres are either blue (mutant) or blue–green (wild type). (D) In an *ire1*^−/−^ whole eye mosaic raised 19°C, an 85% pupal development R cell shows a normal well-organized rhabdomere and ER-containing normal planar rER sheets continuous with abnormal, dilated ER. Scale bars 5 μm (A–C), 1 μm (D).

We asked whether lower temperatures that slow R cell development and reduce biosynthetic demand might mitigate Ire1-loss phenotypes. When grown at 19°C, mutant R cells in mosaic patches were substantially normal (Fig. 7A), whereas dense cytoplasmic NinaA accumulations, SRC loss and small, degenerate rhabdomeres typified mutant R cells of animals grown at 25°C (Fig. 7B). Rh1 levels and rhabdomere delivery were similarly rescued at 19°C (Fig. 7C), and in the electron microscope, ER morphology was substantially more normal (Fig. 7D). Although the rescue was strong, occasional mutant R cells showed deficits at 19°C and some mutant cells appeared normal at 25°C (Fig. S4). In all R cells observed, rhabdomere and ER phenotypes were linked, both were either normal or abnormal, consistent with the simple expectation that normal ER is required to build a normal rhabdomere. Intriguingly, R1 and R6 appeared to be more sensitive than other outer R cells to Ire1 loss. As these cells are born later in the second mitotic wave, it is interesting to speculate R1 and R6 inherit less perdurant cytoplasmic Ire1, relative to the older generation cells.

To determine whether there was a crucial developmental window for Ire1 activity, we systematically shifted developing pupae between 19°C and 25°C and assayed ER and rhabdomere development using confocal microscopy. We found a temperature-sensitive period between ∼48–72% pupal development, at about day 3 of pupal development at 25°C, notably a day before Rh1 expression and the major biosynthetic push that builds the rhabdomere. Animals raised at 19°C during this period had normal ER and rhabdomeres, whereas animals raised at 25°C showed ER and rhabdomere disruption. Ire1 activity thus anticipates peak secretory demand and its loss is manifested as abnormal tangles of tubulated ER as normal cells proliferate stacked rER.

## DISCUSSION

From its initial specification in the morphogenetic furrow, to its adult service as a photosensor, a developing *Drosophila* R cell plays many roles, calling upon a sequence of conserved core cell functions that target the developmental task at hand. In the last quarter of pupal life, that task is the enormous expansion of the photosensory membrane through secretory delivery of membrane rich in rhodopsin and allied elements of phototransduction. Here, we show that a corresponding expansion of stacked rER meets this secretory challenge; at this developmental stage, R cells are typical of cells devoted to intensive secretion, the so-called ‘professional’ secretory cells, generally. As seen with other professional secretory cells, we find that R cells require Ire1 for normal ER expansion and differentiation. When normal cells proliferate rER stacks, Ire1 mutant ER proliferates in a dense and chaotic tangle of reticulon-rich tubules. The mechanisms by which Ire1 supports normal R cell ER differentiation remain unknown and in view of its multiple and far-reaching effectors are likely to be multiple. Shown here recapitulated in R cells, a common theme in Ire1 regulation of ER differentiation is a requirement of anticipation of secretory activity, suggesting that Ire1 builds in secretory capacity as part of normal organelle programming. It is unlikely that Ire1 acts alone and temporal overlap of Ire1 activity, shown here, and also by Coelho et al. (2013), with a second conserved UPR pathway mediated by PERK (Kang et al., 2015) suggests they might cooperate in ER programming. The profuse tangle of reticulon-rich tubules that arises during the peak secretory effort, resembles the pathology seen in Ire1 mutant yeast upon ER stress. There, in response to unfolded protein stress, normal yeast proliferate ER sheets but, although normal in the absence of stress, stressed Ire1 mutant yeast expand ER in dense, reticulon-rich tangles. Taken together, results suggest the possibility that Ire1 contributes essential shaping activity to a program of ER expansion and, in its absence, an unbalanced drive expands dysmorphic ER.

Shown here, and previously (Coelho et al., 2013), Ire1 loss compromises Rh1 production and secretory delivery needed to build the rhabdomere. Prior work has shown that the contribution of Ire1 to these tasks is Xbp1-independent, which we second here and now extend to ER shaping; R cell ER is normal in severe Xbp1 hypomorphs. Previously, it has been shown that Ire1 contributes to Rh1 delivery through degradation of mRNA encoding fatty acid transporter (Fatp): Ire1 loss elevates *fatp* mRNA and its reduction using RNAi rescues Rh1 delivery (Coelho et al., 2013). As elevated levels of phosphatidic acid have been shown to disrupt R cell apical membrane transport (Raghu et al., 2009), it is proposed that increased Fatp elevates phosphatidic acid and thus degrades Rh1 delivery (Coelho et al., 2013). Abnormal, expanded rER is seen in R cells with increased phosphatidic acid (Raghu et al., 2009), but appears to be distinct from the tangled tubular ER seen here. The tubulated ER seen here also differs from the dilated ER lumens commonly noted when misfolded secretory protein products over-accumulate, for example, in Akita mice that accumulate misfolded proinsulin (Zuber et al., 2004), and distinct from the strongly amplified, well-formed rER seen in *ninaA* mutants caused by lumenal Rh1 accumulation (Colley et al., 1991). Failure to assemble expanded rER sheets with accumulation of irregular and ribosome-poor ER tubules in Ire1 mutant R cells is reasonably the ultrastructural reason for the severe reduction of Rh1 levels and growth deficit generally; without a definitive measure of reduced Rh1 synthesis, it is possible that reduced Rh1 levels are attributable to enhanced ERAD in an out of control ER. Loss of a normal SRC in Ire1 mutants plausibly contributes to the failure to deliver secretory traffic to the growing rhabdomere. Collapse of normal ER morphology is thus catastrophic for multiple cell activities.

The mechanism by which lower temperature rescues ER differentiation in Ire1 mutant cells remains to be determined, but might be connected to numerous observations showing that basal, Ire1-independent, ER capacity supports many aspects of normal cell development and physiology. Indeed, despite profound ER disruption during peak rhabdomere growth, mutant R cells are viable and execute a wide range of normal cell physiologies, including normal cell polarity, cell fate specification and the complex choreography that assembles ommatidia. Similarly, Ire1-null nematodes are viable and morphologically normal, but die when challenged with mutations in other UPR branches or tunicamycin (Shen et al., 2001, 2005). Mouse hepatocytes lacking Ire1α show reduced rER content but are otherwise phenotypically normal; however, upon ER stress they fail to maintain lipid homeostasis (Zhang et al., 2011). The gut of mice lacking Ire1β is phenotypically normal, but sensitized to experimental colitis (Bertolotti et al., 2001). In the absence of stress, ER morphology is normal in yeast Ire1 mutants. We speculate that when pupal development is slowed more than twofold (9–10 days at 19°C versus 4 days at 25°C), basal ER capacity meets secretory demand.

The distribution of peripheral ER between sheet and tubule domains has been likened to a ‘tug-of-war’, with activity-promoting ER sheets poised against tubule-promoting activity, particularly that of membrane-curvature-inducing reticulons (Shibata et al., 2010). The near disappearance of rER sheets and concomitant emergence of dense tubular tangles in Ire1 mutants suggests a runaway win for tubule promotion, potentially due to a failure of sheet-forming activities, an abnormal regulation of tubule formation or a combination of both. Ribosomes promote ER sheets and it is possible the loss of ribosomes in mutant R cells destabilizes rER. Alternately, abnormal, dense Rtnl1 accumulations seen in mutant R cells might reflect a cannibalization of ER sheets by misregulated Rtnl1 with a consequent reduction in Rh1 synthesis; misregulated Rtnl1 could also account for the loss of the SRC cortical ER network. The dynamic, stereotyped differentiation of ER morphology underlying *Drosophila* R cell differentiation presents a genetically accessible system to investigate how developmental programs shape ER.

## MATERIALS AND METHODS

### *Drosophila* stocks

Flies were raised on a standard cornmeal diet in a 12-h-light–12-h-dark cycle at 25°C, except during temperature shift experiments. y w; ey FLP; FRT82B GMR-hid/TM2, PBac Ire1^f02170^/TM6B, w ey-FLP;; FRT82B GMR-myr.GFP and FRT42D GMR-hid/Cyo; ey-FLP stocks were obtained from the Bloomington *Drosophila* Stock Center. Ire1^f02170^/TM6B Ubi-GFP and FRT42D Xbp1^k13803^/CyoGFP were provided by Hyung Don Ryoo (New York University, NY). Rtnl1–GFP (Morin et al., 2001) was provided by Lynn Cooley (Yale, New Haven, CT). Mitotic clones in the eye were induced using Flp/FRT with Flippase under the control of the *eyeless* promoter (Xu and Rubin, 1993). Males of the genotype y w; FRT82B Ire1^f02170^/TM6B were crossed to females of the genotype w ey-FLP;; FRT82B GMR-myr.GFP/TM6B. Homozygous Ire1^f02170^ cells lack myristoylated GFP (myr.GFP), whereas heterozygous cells express myr.GFP and appear fully normal. Eyes entirely homozygous for Ire1^f02170^ and Xbp1^k13803^ were generated using the ‘eyeless-Gal4 UAS-FLP’ (EGUF) method (Stowers and Schwarz, 1999). Males of the genotype y w; FRT82B Ire1^f02170^/TM6B or y w; Rtnl1-GFP; FRT82B Ire1^f02170^/TM6B flies were crossed with virgin females of the genotype y w; ey-FLP; FRT82B GMR-hid/TM6B to obtain homozygous Ire1 mutant eyes. Males of the genotype FRT42D Xbp1^k13803^/CyoGFP flies were crossed with virgin females of the genotype FRT42D GMR-hid/Cyo; ey-FLP to obtain homozygous Xbp1^k13803^ mutant eyes.

### Temperature-sensitive period test

Pupal development, taken from white prepupae to eclosion, lasts roughly 218 h at 19°C. To stage pupae, white prepupae were collected and raised to the desired developmental time as a percentage of 218 h. For temperature up-shift experiments, pupae were raised at 19°C and transferred to 25°C at 20% pupal development intervals stage (20, 40, 60, 80 and 100% pupal development) until eclosion. For temperature down-shift experiments we raised pupae at 25°C and transferred them to 19°C at 20% pupal development intervals, taking pupal development at 25°C as 96 h.

### Construction of UPR reporter MXG

*Drosophila* Xbp1 cDNA (GH09250) cloned into pOT2 vector was obtained from the *Drosophila* Genomics Resource Center (DGRC). Given that this cDNA has a deletion of single nucleotide G at the 439th position, site-directed mutagenesis was performed to insert this nucleotide using a kit (Stratagene QuikChange Site-Directed Mutagenesis Kit) with forward primer 5′-GGCCACGCCCTCCGCCTCGCCCACGCCCT-3′ and reverse primer 5′-CGAACTCGAGGGCGTGGGCGAGGCGGAG-3′. Using this cDNA template, the open reading frame (ORF) of unspliced form of Xbp1 (Xbp1u) with the stress specific intron-2 in it, was amplified with primers XBglII-Forward (5′-GGGAGATCTATGGCACCCACAGCAAA-3′) and XEagI-Reverse (5′-GGGCGGCCGATTCAGGCCATTAGCTCTATG-3′) and inserted in between the Myc and GFP constructs in the pUAST vector. This cloning is designed such that GFP is at the C-terminus of Xbp1u and is in the reading frame of Xbp1 only when intron-2 is spliced out by Ire1. A Myc tag present at the N-terminus of Xbp1 reports expression of the construct. Transgenic lines were produced by Rainbow Transgenic Flies, Inc. (Camarillo, CA) using pUAST vectors carrying the MXG construct. Transgenes were expressed with the Gal4-UAS-targeted expression system (Brand and Perrimon, 1993).

### Immunohistochemistry

Dissection of eye and whole mount preparations were performed as described previously (Satoh et al., 2005). Alexa-Fluor-488-conjugated phalloidin for F-actin localization was from Molecular Probes (A-12379, Life Technologies). Rat anti-Elav (1:50 dilution), mouse anti-Rh1 4C5 (1:50 dilution) and mouse anti-Myc 9E10 antibodies (1:50 dilution) were obtained from the Developmental Studies Hybridoma Bank. Rabbit anti-Rh1 N-terminal antibody (1:1000 dilution) was made by our laboratory (Satoh et al., 2005). Mouse monoclonal anti-GFP (clone 3E6) antibody was obtained from Life Technologies. Rabbit polyclonal anti-NinaA was a gift from Nansi Jo Colley (Department of Ophthalmology and Visual Sciences and Department of Genetics, University of Wisconsin, Madison, WI). FM4-64 for membrane staining was from Invitrogen (F34653). Secondary antibodies were anti-mouse and -rabbit-IgG, labeled with Alexa Fluor 488, 568 or 647 (Molecular Probes). Samples were mounted onto glass slides with mounting medium containing 50% glycerol in PBS and 0.25% n-propyl gallate (pH 7.5), and images were taken using a Zeiss LSM 710 confocal microscope.

### Electron microscopy and electron tomography

Flies were prepared for transmission electron microscopy essentially as described in Longley and Ready (1995) with modifications. Ultrathin sections were cut with a diamond knife using a Reichert-Jung Ultracut microtome. Sections were stained with Reynold's lead citrate and 2% aqueous uranyl acetate and were observed with a Philips CM-100 transmission electron microscope. For electron tomography, 420 nm thick sections were cut and a 200 kV Tecnai G2 20 electron microscope (FEI) equipped with a Gatan CCD camera was used to record serial tilts of ±60° in increments of 1°. Tomogram reconstruction was carried out using IMOD software (Kremer et al., 1996).

### Ommatidium cross-section area measurement

Six Ire1 mosaic retinas from six 1-day-old adults were selected. Two were examined using electron miscopy and four were examined using confocal microscopy. ImageJ was used to measure ommatidial cross-sections at ∼10–20 μm below the cone; each field contained a mosaic patch with 4–10 Ire1 mutant and 7–12 wild-type ommatidia. The data are presented as mean±s.d. For each mosaic field, a Student's *t*-test was used to compare the cross-sectional area difference between mutant and wild-type ommatidia. *P*<0.05 was considered to be statistically significant.

### Ommatidium isolation and length measurement

Adult *Drosophila* ommatidia were prepared as described previously (Hardie et al., 1991). Briefly, retinas expressing Rtnl–GFP were rapidly dissected in M3 insect medium (Sigma), transferred to M3 medium supplemented with 10% serum, and gently triturated using a fire polished micropipette, then transferred to a Sigmacote SL-2 treated coverslip with the above medium. Dissociated ommatidia were gently separated using a fine dissected needle. Images were taken immediately using a Zeiss LSM710 confocal microscope. Ommatidial length was measured using ImageJ.
